# Ultrasound Utility in the Diagnosis of a Morel-Lavallée Lesion

**DOI:** 10.1155/2017/3967587

**Published:** 2017-02-01

**Authors:** Scott LaTulip, Rameshwar R. Rao, Alan Sielaff, Nik Theyyunni, John Burkhardt

**Affiliations:** ^1^University of Michigan Medical School, Ann Arbor, MI 48109, USA; ^2^Emergency Physicians Medical Group, 2000 Green Road No. 300, Ann Arbor, MI 48105, USA; ^3^Department of Emergency Medicine, University of Michigan Health System, Ann Arbor, MI 48109, USA; ^4^Departments of Emergency Medicine and Learning Health Sciences, University of Michigan Health System, Ann Arbor, MI 48109, USA

## Abstract

Morel-Lavallée lesions are uncommon injuries that can be associated with significant comorbidities if not detected early. Rapid diagnosis in the Emergency Department could significantly improve patient outcomes. We describe the diagnosis of such a lesion through the use of ultrasound imaging in the Emergency Department to utilize a fast, cost-effective imaging technique that does not subject the patient to radiation exposure. Our patient received surgical consultation but improved with conservative management. Ultrasound findings associated with this lesion do not require specialized equipment and should be considered when evaluating soft tissue lesions using point of care ultrasound.

## 1. Introduction

A Morel-Lavallée (ML) lesion is an internal degloving injury which separates tissue layers along their fascial planes. These lesions are relatively rare and most often occur secondary to trauma. This lesion was first described in the medical literature in 1863 by a French surgeon named Morel-Lavallée [1]. It is believed that ML lesions arise in certain traumatic situations when a predominantly shearing force is sustained to the soft tissue. This shearing force results in a separation of the skin and subcutaneous adipose tissue from the underlying fascial plane, creating a potential space which can then become filled with blood, lymph, and serous fluid.

Missed diagnosis of a ML lesion can potentially result in numerous additional specialist referrals and imaging studies, delayed treatment, and poor functional outcomes. The gold standard for diagnosis of a Morel-Lavallée lesion is an MRI [2]. In certain cases, initial diagnosis with ultrasound can be just as effective, while also being a more rapid and low-cost option. The following case exemplifies how easily a ML lesion can be missed on initial presentation, as well as the utility of ultrasonography in diagnosing this condition.

## 2. Case Presentation

A 20-year-old, otherwise healthy, male presented to the Emergency Department (ED) after falling on his back while playing basketball. The patient landed on his right buttock on the hardwood floor. On presentation to the ED, he denied back pain, numbness, and weakness but did notice progressive swelling in the area of the fall. He denied any other injuries and had no previous bleeding or bruising history. His medical history was unremarkable for bleeding disorders or anticoagulation. He denied previous issues with hematomas, subcutaneous fluid collections, or abscesses.

On physical exam, the patient was not in acute distress and showed equivalent and intact strength and sensation in all four extremities. There was no weakness, numbness, or tingling in the lower extremities. All distal pulses were equal and palpable. The patient had mild pain in the right gluteal region with impressive swelling in the area. There was no lumbar, sacral, or pelvic tenderness to palpation. Point of care ultrasound demonstrated an 8 × 2.8 cm^2^ fluid collection in the right gluteal region which was incorrectly thought to be located within the muscle belly. He was given an ice pack, instructed to use Tylenol, and given a referral to Orthopaedics. He was instructed to return to the ED if the pain worsened or if he developed fevers, chills, nausea, vomiting, or other symptoms and was discharged the same evening.

The patient returned to the ED four days later for worsening pain and swelling of his buttock. He was unable to tolerate running and had worsening pain upon walking. The patient was concerned for a possible abscess at the injured area as it had become increasingly tender to touch since his previous visit to the ED. The skin at the area was intact and the patient denied drainage. He did not present with any constitutional symptoms including fevers, chills, nausea, vomiting, abdominal pain, lightheadedness, dizziness, or syncope. Physical exam was positive for myalgia in the right upper gluteal region. A complete blood count (CBC) revealed mildly microcytic RBCs of 78.3 fL (normal = 79.9–99.0), decreased mean corpuscular hemoglobin of 26.5 fmol/cell (normal = 27.0–32.0), and a decreased monocyte percentage of 5.8 (normal = 6.0–13.0). White blood cell count was 6.3 × 10^3^ cells/mL (normal = 4.0–10.0), hemoglobin was 13.7 gm/dL (normal = 13.5–17.0), RBC count was 5.17 × 10^3^ cells/mL (normal = 4.40–5.70), platelet count was 228 × 10^3^ cells/mL (normal = 150–400), platelet thromboplastin time (PTT) was 25.1 seconds (normal = 22.0–32.0), prothrombin time (PT) was 10.9 seconds (normal = 9.5–12.0), and International Normalized Ratio (INR) was 1.0.

**Figure 1:**
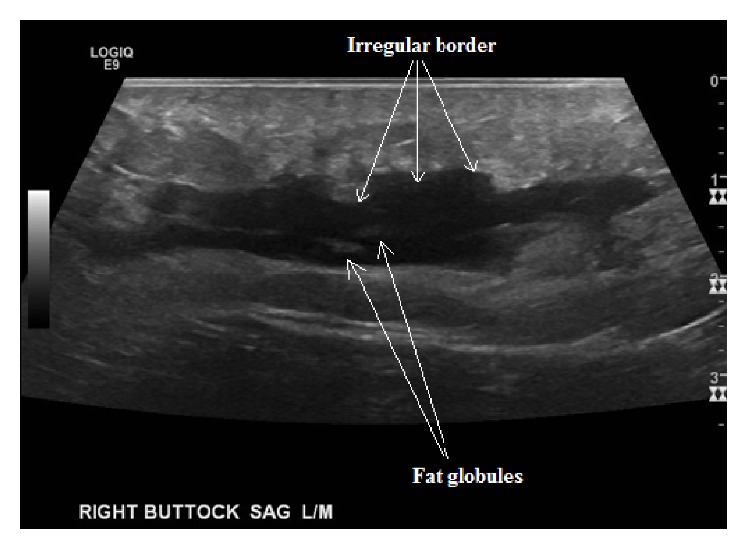
Sagittal ultrasound view of lesion. Note irregular borders and echogenic fat globules.

**Figure 2:**
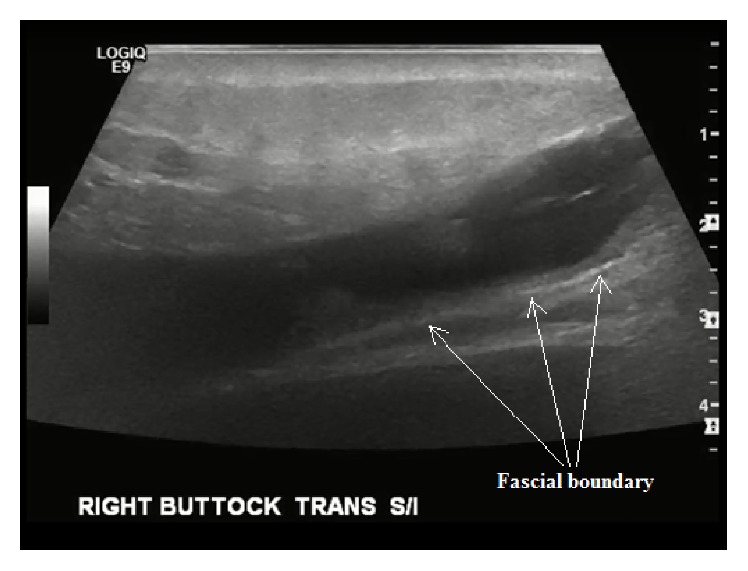
Transverse ultrasound view of lesion. Note fluid collection bounded by fascial planes.

A radiology-based musculoskeletal ultrasound was performed on the right upper gluteal region. The exam was notable for a large hypoechoic fluid collection deep to the subcutaneous tissue and superior to the gluteal musculature (Figures 1 and 2). The fluid collection had increased to 13 × 2 × 9 cm^3^. Internal echoes and fat globules were found within the hypoechoic fluid collection. These findings were suggestive of a Morell-Lavallée closed degloving lesion. Plastic Surgery was consulted and opted not to perform drainage of the lesion as there was no superficial skin breakdown. The patient was given a pressure dressing for the lesion and recommended to follow up with Plastic Surgery clinic for further management of the lesion.

## 3. Discussion

The Morel-Lavallée lesions can have a highly variable clinical presentation. Presenting symptoms including pain and swelling, symptoms mimicking DVT, and decreased sensation secondary to damage to cutaneous nerves have all been described [3]. These lesions can develop anywhere from hours to several weeks after trauma sustained to the region [4]. Other common conditions with a similar presentation include hematomas and sarcomas. Prompt diagnosis of ML is important as infection rates have been cited from 19% to 46% [4, 5]. To make the diagnosis, an emergency physician must maintain a high clinical suspicion for this diagnosis; however, this can be facilitated by the early use of point of care ultrasound [4]. While MRI may be the diagnostic test of choice for a Morel-Lavallée lesion, ultrasound findings combined with a high clinical suspicion can lend themselves to the diagnosis [2].

The prognosis for a ML lesion can range from spontaneous resolution over a few weeks to encapsulation and chronic persistence. Initial Emergency Department treatment is typically conservative with compression wrap, range of motion exercises, and referral for specialist consultation. Treatment of persistent or recurrent lesions can include aspiration, sclerodesis with various agents including antibiotics, or surgical drainage in more severe cases [6]. Case reviews have shown initial miss rates of greater than 40% of ML lesions on presentation [7]. This delay in diagnosis then leads to an increased need for surgical debridement [8].

Previous studies on ML lesions have described fluid collections between the deep fat and fascial layers (Figure 2). The fluid collections can have atypical contours and often contain debris and fat globules within the fluid (Figure 1). Noting these atypical features and confirming the correct plane (muscle versus subcutaneous) are critical to missing this diagnosis on bedside ultrasound. Unfortunately, findings depend significantly on the age of the lesion [9–11]. Neal et al. described the ultrasonographic findings in a Morel-Lavallée lesion with respect to age [12]. They found that most were homogenous in appearance and with respect to echogenicity; the majority were found to be hypoechoic or anechoic. They also concluded that lesions less than two weeks were lobular in appearance and those greater than six months tended to have a flat appearance. Furthermore, muscle contusion or laceration can occasionally be noted on ultrasound to help support the diagnosis [13].

This case report describes a Morel-Lavallée lesion that presented both in an unusual location and with a somewhat atypical mechanism. More importantly, it also describes the first description of the potential use of bedside ultrasonography in the evaluation of a suspected Morel-Lavallée lesion. Notably, in this case, the initial team using point of care ultrasound did not make the diagnosis of ML lesion. Being aware of the diagnosis is as important as knowing the typical sonographic features. This lesion appears as a fluid collection adjacent to subcutaneous tissue and muscle and thus may be confused for fluid collections within these locations, such as an abscess, a simple hematoma, or a contusion within a muscle.

For point of care providers to find acute Morel-Lavallée lesions, they must know their specific appearance: lobular appearance, adjacent disruption of fascial planes, and/or internal echogenic debris and fat globules. Use of both a high frequency linear probe and a lower frequency curvilinear probe also has benefit as a high frequency probe is more likely to demonstrate the fat globules, internal debris, or the irregular borders of the lesions. Using graded compression on the lesion can also help differentiate an ML lesion from abscess, as abscesses often have a fairly classic appearance with compression sonography; however, this technique may be limited by patient comfort.

Our case highlights classic ultrasound findings consistent with the Morel-Lavallée lesion. Use of point of care ultrasonography by Emergency Physicians may be a cost-effective way to increase initial accuracy in making this diagnosis. Expedited treatment and management may mitigate late occurring complications such as infection and abscess formation.
